# “I Will Never Be Enough Because My Skin Color Isn’t Right”: Structural Racism and Healthcare Access Following Nonfatal Strangulation Among Black Women Survivors of Intimate Partner Violence

**DOI:** 10.3390/healthcare14183007

**Published:** 2026-09-14

**Authors:** Jeneile Luebke, Kaylen M. Moore, Lucy Mkandawire-Valhmu, Leso Munala, Hanan Abusbaitan, Frances Kimber, Jacqueline Callari Robinson, Breanna Heisterkamp, Antonia Norton, Anna Pirsch, Peninnah M. Kako, Alexa Lopez

**Affiliations:** 1 School of Nursing, University of Wisconsin, Madison, WI 53705, USA; jmluebke@wisc.edu; 2 College of Nursing, Marquette University, Milwaukee, WI 53233, USA; kaylen.moore@marquette.edu; 3 School of Nursing, University of Minnesota, Minneapolis, MN 55455, USA; lmkandaw@umn.edu (L.M.-V.); lmunala@umn.edu (L.M.); fulle360@umn.edu (B.H.); 4 School of Nursing, Oakland University, Rochester, MI 48309, USA; habusbaitan@oakland.edu; 5 School of Nursing, University of Wisconsin, Milwaukee, WI 53201, USA; callari2@uwm.edu (J.C.R.); pmkako@uwm.edu (P.M.K.); andersoa@uwm.edu (A.L.); 6 Asha Family Services, Milwaukee, WI 53210, USA; antonian@ashafamilyservices.org; 7 Department of Nursing, Augsburg University, Minneapolis, MN 55454, USA; pirsch@augsburg.edu

**Keywords:** intimate partner violence, strangulation, Black women

## Abstract

**Background**: Nonfatal strangulation (NFS) is one of the most lethal forms of intimate partner violence (IPV) that is associated with increased risk of future homicide, yet it frequently goes unrecognized within healthcare settings. Black women experience disproportionately high rates of IPV while simultaneously facing structural barriers to healthcare access. Little research has examined how Black women survivors experience strangulation and navigate decisions about seeking care following NFS and other potentially lethal forms of IPV. **Methods**: This secondary qualitative analysis draws on interview data from 28 Black women survivors of IPV who participated in a larger community-engaged mixed-methods study conducted during the COVID-19 pandemic in an Upper Midwestern state. Of these, 17 (60.7%) both reported experiencing strangulation on the parent-study survey and described experiences consistent with nonfatal strangulation during their qualitative interviews and comprised the analytic sample for the present analysis. Guided by Black Feminist Thought, semi-structured interviews examined survivors’ experiences with violence, safety, help-seeking, and interactions with healthcare and social service systems. Data were analyzed using thematic analysis following Schensul and Schensul. **Results**: Three interconnected themes were identified. First, survivors described racism and anticipated discrimination as significant barriers to seeking healthcare following strangulation. Second, shame, stigma, and fear of judgment contributed to self-silencing and avoidance of formal services. Third, survivors described strangulation as a terrifying and potentially lethal form of violence occurring within broader histories of polyvictimization and cumulative trauma. Despite experiencing serious symptoms, many participants avoided healthcare because they feared retaliation from abusive partners, anticipated dismissive treatment, or believed healthcare systems would be unable to protect them. Together, these experiences illustrate how structural racism, institutional mistrust, and gendered violence intersect to shape healthcare decision-making. **Conclusions**: Findings suggest that barriers to care following strangulation extend beyond individual help-seeking behaviors and are deeply rooted in structural inequities. Improving outcomes for Black women survivors requires enhanced provider training on strangulation assessment, trauma- and violence-informed approaches, culturally responsive care, and healthcare system reforms that address racism, mistrust, and survivor safety. These findings have important implications for reducing disparities in IPV-related health outcomes and improving responses to high-lethality violence.

## 1. Introduction

Nonfatal strangulation (NFS) is a highly lethal form of intimate partner violence (IPV) characterized by external compression of the neck that restricts blood flow, airflow, or both without resulting in death [1]. NFS in intimate partner relationships is often used as a coercive tactic of control, intimidation, and terror, with the act itself functioning not only as a means of physical harm but also as a demonstration of dominance intended to instill fear, reinforce power imbalances, and restrict a survivor’s autonomy and ability to seek help [2]. Despite its life-threatening nature, NFS is frequently underrecognized in clinical settings due to the absence of visible external injury and survivors’ impaired recall and cognitive symptoms following the event, which may hinder disclosure and complicate clinical identification even in the presence of significant internal and neurological harm [3,4]. Empirical studies demonstrate that survivors often present to healthcare settings without overt physical findings despite reporting severe symptoms such as loss of consciousness, neurologic changes, voice or swallowing difficulties, and respiratory distress [5,6]. This clinical invisibility contributes to missed opportunities for identification and intervention, even when significant internal and neurologic injury is present.

NFS is associated with serious acute and long-term health consequences, including neurological and psychological sequelae such as anoxic and traumatic brain injury, cerebrovascular complications, cognitive and memory impairment, executive dysfunction, difficulties with concentration, vision disturbances, anxiety, depression, and post-traumatic stress disorder, and is increasingly understood as a sentinel indicator of escalating violence and heightened risk for subsequent lethality [3,7,8]. Research has demonstrated that women with a history of IPV-related strangulation may experience persistent neurobehavioral symptoms and traumatic stress years after the strangulation event, suggesting that the effects of strangulation may extend well beyond the acute injury period [9]. Furthermore, repeated episodes of strangulation may produce cumulative neurological damage through recurrent oxygen deprivation and cerebrovascular injury, potentially contributing to chronic cognitive impairment and reduced functional capacity. These consequences may also contribute to acquired disability following NFS, while survivors living with pre-existing disabilities may face additional barriers to accessing healthcare, communicating their experiences, and having their reports of violence recognized and believed.

In response to this growing evidence base, many jurisdictions have enacted strangulation-specific statutes and enhanced criminal penalties to improve recognition and accountability within the legal system [10]. Collectively, these developments underscore NFS as both a medical emergency and a critical forensic concern requiring timely recognition, accurate documentation, and coordinated intervention within healthcare and criminal justice systems. Despite this growing attention, less is known about how Black women survivors of IPV experience healthcare responses following NFS and how these encounters shape disclosure, trust, and access to care. Centering these lived experiences is essential to identifying gaps in clinical recognition and response, and to informing more equitable, trauma-informed approaches to the identification and management of patients at risk for severe and potentially lethal violence.

### 1.1. Literature Gap: Black Women and Nonfatal Strangulation

Despite growing recognition of NFS as a significant predictor of intimate partner homicide and long-term health consequences, little research has examined the experiences of Black women survivors [11]. The existing literature largely treats women as a homogeneous group, failing to account for the intersecting influences of race, gender, structural racism, healthcare inequities, and criminal justice disparities [12]. Consequently, little is known about how Black women experience, disclose, seek care for, and recover from NFS, or how healthcare and forensic systems respond to their needs. An intersectional, survivor-centered examination is needed to inform culturally responsive forensic nursing practice, policy, and violence prevention efforts.

### 1.2. Lack of Understanding of Strangulation Language and Terminology

Another significant gap in the literature is the limited understanding of how survivors describe strangulation experiences and how terminology influences assessment, disclosure, documentation, and clinical decision-making. Researchers, healthcare providers, law enforcement personnel, and legal professionals frequently use the term “strangulation” to describe external compression of the neck, while survivors often use terms such as “choking,” “choked,” “grabbed my neck,” “couldn’t breathe,” “put his hands around my throat,” or other descriptive language that may not align with medical or forensic terminology [10].

The distinction between strangulation and choking is clinically important. In medicine, choking refers to an internal airway obstruction, whereas strangulation refers to external pressure applied to the neck that compromises airflow, blood flow, or both. However, survivors frequently use the term “choking” when describing a strangulation event, and providers may fail to recognize the lethality implications if they rely solely on clinical terminology rather than exploring the patient’s narrative [13,14].

### 1.3. Violence in the Lives of Black Women

Black women in the United States face disproportionately high rates of IPV, sexual assault, and homicide compared to White women [15,16]. Despite these disparities, Black women remain underrepresented in research and continue to encounter structural barriers to equitable healthcare and culturally responsive post-assault services [17,18]. Structural racism, discriminatory medical practices, and historical trauma heavily influence Black women’s access to care. The persistent racist stereotype that Black women are “unrapeable” originates in slavery, when Black women were denied bodily autonomy and legal protections, and rape was normalized and legally sanctioned [19,20]. These legacies continue to shape provider perceptions, clinical decision-making, and institutional responses to violence against Black women [21].

Racial bias in healthcare leads to misdiagnosis, undertreatment of pain, disbelief in Black women’s reports of violence, and failure to identify or respond to injuries from strangulation and other forms of IPV [22,23]. Black women often expect judgment, criminalization, and disrespect when interacting with healthcare providers, which may contribute to delays in seeking care or avoidance of medical care after violence [17,24]. These experiences are compounded by negative encounters with law enforcement, child welfare systems, emergency departments, and shelters, which often reinforce feelings of shame, isolation, and the belief that services are not meant for Black women [25].

Structural racism encompasses interconnected systems and institutional practices that shape the distribution of resources, opportunities, and health risks across racial groups [26]. The COVID-19 pandemic further illuminated these structural inequities for Black women experiencing IPV, as pandemic-related disruptions and economic instability intersected with existing structural barriers to help-seeking, healthcare, and other forms of support [27]. Increased isolation, financial stress, and limited access to advocacy and healthcare services led to higher severity of IPV, including strangulation [28,29]. For Black survivors, these risks were even greater due to long-standing health inequalities, racial disparities in COVID-19 impact, and the disproportionate policing and surveillance of Black communities during the pandemic [30]. In addition, help-seeking behaviors were further constrained by reduced availability of culturally responsive services and increased barriers to accessing formal support systems, particularly for Black and Indigenous women whose engagement with healthcare and advocacy services was shaped by structural racism and mistrust of institutions [31]. Shelter-in-place orders limited movement and privacy, trapping many survivors with abusive partners and preventing safe contact with healthcare providers or advocates.

Guided by Black Feminist Thought, this study centers the lived experiences of Black women who survived IPV during the COVID-19 pandemic in an upper Midwestern state. This theoretical framework emphasizes relationality, community accountability, and survivor expertise. It also highlights how intersecting forms of oppression, such as racism, sexism, poverty, and criminalization, affect survivors’ experiences with healthcare and forensic systems [14,19]. By focusing on women’s narratives, this study explores how systemic racism, shame, and ongoing trauma create barriers to accessing medical-forensic care, even when injuries resulting from strangulation are severe. Recognizing these barriers is vital for improving culturally responsive support services, forensic nursing practices, and outcomes for Black survivors of IPV.

The parent study sought to understand Black and Indigenous women’s experiences with intimate partner violence, help-seeking, and access to support services during the COVID-19 pandemic. During analysis, narratives of NFS recurred across interviews and were identified as a critical focus because of the serious health consequences and elevated homicide risk associated with this particular form of IPV. The aim of the present analysis was to examine how Black women survivors described experiences of NFS and how structural racism, shame, and institutional mistrust shaped their decision to seek healthcare following this lethal form of IPV. Guided by this aim, we examined:

1. How Black women survivors described nonfatal strangulation as a form of IPV they experienced.
2. How racism and shame shaped women’s decisions to seek or avoid healthcare following strangulation.

## 2. Materials and Methods

### 2.1. Study Design and Context

This study presents a secondary qualitative data analysis of interview data collected as part of a larger community-engaged mixed-methods R01 study examining IPV, help-seeking behaviors, and perceived barriers to support among Black and Indigenous women during the COVID-19 pandemic. The parent study was designed to investigate how structural, interpersonal, and economic conditions shaped survivors’ experiences of violence and their access to support during a public health crisis. The broader study prioritized survivor voice, community knowledge, and relational accountability. These principles were reinforced through collaboration with a community advisory board (CAB), which provided guidance on study procedures, community priorities, violence prevention efforts, and public health recommendations. Detailed descriptions of the broader study design, recruitment procedures, and CAB structure have been published elsewhere [32,33].

The present analysis focuses specifically on Black women survivors of IPV and their experiences with NFS and healthcare access. Although the parent study was not designed specifically to examine strangulation, narratives describing strangulation were identified repeatedly across interviews. Given the significant health consequences, elevated risk of future homicide, and barriers to healthcare associated with nonfatal strangulation, this prominent pattern within the broader qualitative dataset was explored in greater depth in the present analysis.

Although the present manuscript focuses on qualitative interviews with Black women who described NFS, preliminary descriptive findings from the broader mixed-methods parent study also underscore the significance of this issue. Among the 232 Black and Indigenous women who completed the parent study survey, 186 (80%) reported that a current or former partner had ever attempted strangulation. Together, the prominence of strangulation in survivors’ narratives and its high prevalence in the broader cohort underscore the importance of understanding Black women’s experiences after NFS. Additional quantitative and mixed-methods analyses from the parent study are forthcoming and will further examine the prevalence, correlates, and health consequences of NFS in this cohort.

### 2.2. Theoretical Framework

This analysis was guided by Black Feminist Thought (BFT), which centers the lived experiences and knowledge of Black women and emphasizes that violence against Black women must be understood within intersecting systems of racism, sexism, and other forms of oppression [19,21]. Collins’ concept of the matrix of domination highlights how intersecting systems of oppression collectively shape Black women’s experiences and the options available to them. This intersectional perspective is particularly important for understanding IPV within the broader racial, gendered, socioeconomic, and structural conditions that shape Black women’s lives [34].

Applied to IPV and healthcare access, BFT provides a framework for understanding how Black women’s decision to seek healthcare following nonfatal strangulation may be shaped by multiple, intersecting conditions. Research with Black women experiencing IPV has similarly shown that help-seeking occurs within complex racialized, gendered, and socioeconomic contexts [11]. Structural racism and prior institutional experiences may shape how survivors anticipate being treated within healthcare and other systems, while socioeconomic disadvantage may constrain available resources and options, and ongoing IPV may create immediate concerns about safety and retaliation. Although these barriers are distinct, they may intersect in ways that shape women’s decisions about whether and when they seek care.

BFT also centers Black women’s agency and resistance within these structural conditions [19]. From this perspective, delaying or avoiding healthcare following NFS should not necessarily be understood as a failure to recognize the seriousness of strangulation or as an individual deficit in help-seeking. Rather, women’s decisions may reflect assessments of the potential risks and benefits of seeking care within the context of prior institutional experiences, available resources, and ongoing concerns about safety. BFT therefore guided our interpretation of Black women’s narratives about NFS and healthcare access while maintaining attention to both women’s agency and the intersecting structural conditions surrounding their decisions.

### 2.3. Positionality

The research team brought together diverse disciplinary and lived perspectives and included feminist, anti-violence, and forensic nurse scholars with expertise in intimate partner violence, qualitative methods, trauma-informed care, and health equity. Members of the team identified as Indigenous, Black, Hapa, Middle Eastern, and White women and brought disciplinary perspectives from nursing, public health, and social work. These varied social locations and professional experiences informed our engagement with the data and interpretation of participants’ narratives. Throughout the analytic process, team members engaged in reflexive dialogue to examine assumptions, challenge interpretations, and ensure that findings remained grounded in participants’ narratives.

### 2.4. Participants and Recruitment

Participants were recruited as part of the larger parent study examining IPV, help-seeking, and access to support services among Black and Indigenous women during the COVID-19 pandemic. Eligibility for the parent study was based on participants’ experiences of IPV rather than experiences of nonfatal strangulation specifically. The qualitative dataset included 28 participants who self-identified as Black women and reported experiences of IPV. Of these, 17 (60.7%) both reported experiencing strangulation on the parent-study survey and described experiences consistent with nonfatal strangulation during their qualitative interviews. These 17 participants comprised the analytic sample for the present focused secondary analysis.

Participants were recruited through community-based organizations, domestic violence advocacy programs, and existing community partnerships using purposive and snowball sampling. Recruitment procedures were developed in collaboration with the study’s CAB and prioritized participant safety and confidentiality. Women were recruited from an urban region marked by persistent racial inequities, concentrated poverty, and disproportionately high rates of IPV-related homicide among Black women.

### 2.5. Data Collection

Semi-structured interviews were conducted between 2022 and 2025 by trained members of the research team. Interviews lasted 60 to 90 min and were conducted in person, by telephone, or via secure virtual platforms, based on participant preference and safety considerations.

The interview guide was developed collaboratively with members of the research team and community partners to explore survivors’ experiences with intimate partner violence, safety, help-seeking, and interactions with healthcare, legal, and social service systems during the COVID-19 pandemic; Therefore, participants shared their retrospective accounts of pandemic-era violence and help-seeking. Questions were open-ended and participant-centered, allowing women to describe their experiences in their own words while maintaining consistency across interviews. Interview topics included conceptualizations of safety, experiences of violence, barriers to support, and help-seeking from formal and informal sources.

Because NFS was not a primary focus of the parent study, the interview guide did not systematically elicit the timing of individual strangulation events. Although participants were recruited based on experiences of IPV during the COVID-19 pandemic, their narratives sometimes extended beyond the pandemic period and included both recent and more temporally distant experiences of violence. Consequently, the timing of NFS relative to the interview could not be consistently determined across participants.

Interviews were audio-recorded with participant consent, professionally transcribed verbatim, and de-identified before analysis. Although strangulation was not a primary focus of the interview guide, experiences of NFS emerged repeatedly during discussions of violence, safety, and healthcare access. The frequency, severity, and potential lethality of these accounts prompted a focused analysis of Black women’s experiences of strangulation and barriers to healthcare access.

Community engagement remained central throughout the research process. Community partners and CAB members informed study procedures and contributed to discussions on interpreting findings to enhance contextual relevance and ensure that survivor perspectives remained central to the analysis.

### 2.6. Data Analysis

Data were analyzed thematically using an inductive qualitative approach informed by Schensul and Schensul [35]. Analysis began with repeated reading of the interview transcripts to develop familiarity with participants’ accounts and identify initial patterns relevant to the broader study questions. Initial codes were generated from participants’ narratives and organized into related categories and broader patterns of meaning. Coding remained grounded in participants’ descriptions of their experiences rather than being structured around a predetermined set of themes.

During analysis of the broader qualitative dataset, participants repeatedly described experiences of being “choked”, “strangled,” or otherwise having pressure applied to the neck or throat by an intimate partner. Given the recurrence and severity of these accounts and the serious health consequences and elevated lethality associated with NFS, these narratives became the focus of the present secondary analysis. The relevant transcripts were revisited to examine these accounts in greater depth. Coding related to NFS, healthcare access, racism, shame, safety, and responses to violence was examined across participants’ accounts and organized into broader patterns and candidate themes. Themes were iteratively reviewed and refined by examining how well they represented the coded data and the broader narratives from which those data were drawn.

Analysis and theme development were reviewed by members of the research team, who discussed the organization and interpretation of the findings and refined themes to maintain coherence within and distinction across them. Excerpts selected to illustrate the findings were verified against the original transcripts. This iterative process resulted in three primary themes that captured participants’ experiences of NFS and the conditions shaping healthcare decision-making following these experiences.

Black Feminist Thought informed the interpretive phase of the analysis by directing attention to how racialized, gendered, socioeconomic, and structural conditions shaped women’s experiences of NFS and healthcare access. Rather than treating racism, socioeconomic circumstances, institutional mistrust, and IPV-related safety concerns as interchangeable barriers, the interpretive analysis considered how these distinct conditions operated and intersected within participants’ accounts. BFT also directed attention to women’s agency and to how decisions to seek, delay, or avoid healthcare could reflect assessments of safety and potential institutional harm within the structural contexts surrounding their lives.

### 2.7. Reflexivity and Rigor

Methodological rigor was supported through several strategies designed to strengthen the trustworthiness of the analysis. Credibility was supported through repeated engagement with the interview transcripts, iterative review and refinement of the analysis, and review of the developing themes by members of the research team. Dependability was supported through an iterative analytic process in which codes, patterns, and themes were revisited and refined as the analysis developed. Confirmability was strengthened by grounding interpretations in participants’ narratives, reviewing the analysis among members of the research team, and verifying excerpts selected to illustrate the findings against the original transcripts. Transferability was supported through detailed description of the study context, participant characteristics, recruitment and data collection procedures, and analytic approach, allowing readers to assess the relevance of the findings to other populations and settings.

### 2.8. Ethical Considerations

Our study received approval from the appropriate institutional review board. All participants provided informed consent before participating. To support emotional well-being, safety protocols were developed in collaboration with community partners and domestic violence advocates. Participants were reminded that they could decline to answer any question or stop the interview at any time. Referrals to culturally specific advocacy and support services were provided as needed. Additional details on recruitment and safety procedures have been reported elsewhere [32,33].

## 3. Results

### 3.1. Participant Characteristics

Among the 28 Black women in the qualitative dataset, 17 (60.7%) comprised the analytic sample for the focused NFS analysis. Participants were 37.18 years of age (SD = 9.63), with a range of 20–54 years. Among participants who reported educational attainment, over half had completed high school as their highest level of education (53.6%), and half of the participants were unemployed (50.0%). Among participants who reported annual household incomes, most reported incomes below $20,000, including assistance, reflecting the substantial economic disadvantage among respondents with available income data. As seen in Table 1, some participants did not respond to all demographic questions, with about one-third of participants not providing income information. Additional demographic characteristics are presented in Table 1.

### 3.2. Findings

Through thematic analysis, we identified three primary themes describing the experiences of the 17 women included in the focused NFS analysis, including their experiences accessing healthcare following IPV that involved NFS, which women described as being strangled or choked. The analysis focuses solely on the narratives of women who had survived strangulation, a uniquely lethal form of assault. The first two themes, “The scum of the earth”: Blackness as a Barrier to Accessing Care After Strangulation and “It was embarrassing”: Shame as a Barrier to Help-Seeking Following Strangulation, detail the contextual barriers of racism and shame that shaped how women responded to strangulation. The third theme, “When you’re trying to get your breath back, it’s so horrible”: Severe Injury and the Unique Trauma of Strangulation, details the severe physical and psychological reality of the strangulation itself, which simultaneously created an urgent need for care and presented profound obstacles to seeking it.

#### 3.2.1. “The Scum of the Earth”: Blackness as a Barrier to Accessing Care After Strangulation

For Black women who had survived strangulation, their racial identity was a primary determinant in deciding not to seek medical care, even for potentially life-threatening injuries. This theme comprises two subthemes, (1) anticipated racism and dehumanization and (2) strategic self-reliance as a survival tactic, describing interlocking dynamics women experienced.

Anticipated racism and dehumanization: Women internalized a belief that they would be deemed unworthy of care due to racism, making healthcare access feel pointless after an assault. One woman explained:

*“They look at Black people like we’re the scum of the earth… It makes you feel like not enough; I will never be enough because my skin color isn’t right.”*

Strategic self-reliance as a survival tactic: In response to this anticipated mistreatment, women adopted a posture of extreme self-reliance. Having learned from past institutional failures (healthcare, parole), they prioritized avoiding systems altogether, even for injuries from strangulation. One participant articulated this calculated stance:

*“Majority of my life, I know what to do, I know how to calm down… if I don’t have to call police or take those measures… I’ll figure it out.”*

When asked directly if she would seek care for physical injuries, her response was definitive:

*“No, I don’t… if I don’t have to, I won’t.”*

For her, the potential for institutional harm deterred healthcare seeking, even when weighed against the serious medical risks associated with strangulation.

#### 3.2.2. “It Was Embarrassing”: Shame as a Barrier to Help-Seeking Following Strangulation

The act of strangulation, an intensely violent form of control, generated profound shame that was compounded by intersecting experiences. Two subthemes, (1) public exposure and humiliation and (2) internalized stigma and fear of judgement, showed how the shame directly inhibited disclosure and help-seeking through two key mechanisms:

Public exposure and humiliation: The process of seeking help often involved a humiliating lack of privacy, which retraumatized women and deterred future help-seeking. One woman described the shame of having to access help from law enforcement publicly:

*“It was embarrassing… when I had called the police on him… they actually came and took my pictures outside in front of people… that was an experience that was very embarrassing to me.”*

Internalized Stigma and fear of judgement: Shame was also internalized, shaped by anticipated community judgement and the stigma associated with being a survivor of IPV, leading to self-silencing and reluctance to disclose strangulation. One participant explained:

*“Yeah, I didn’t really engage or tell someone because I feel like they’ll say someone else or they…be hearing it from someone else … have to confide in someone is going to be hard, that’s just—I mean, I just—you know, keep it in.”*

This shame extended to accessing shelters, making the simple act of walking through a door feel insurmountable after what women described as one of the most degrading forms of violence.

#### 3.2.3. “When You’re Trying to Get Your Breath Back, It’s So Horrible”: Severe Injury and the Unique Trauma of Strangulation

This third primary theme details the acute reality of strangulation and the intertwined reasons women did not seek care for these specific injuries. Women described strangulation as a distinct, terrifying escalation of violence that created a paralyzing conflict between an urgent need for care and an overwhelming set of barriers to accessing it. This primary theme yielded five subthemes that described women’s physical and psychological realities of NFS: (1) fear of lethal retaliation, (2) perceived system futility, (3) normalization and minimization of lethal risk, (4) trauma of recall, and (5) polyvictimization and cumulative trauma.

Fear of lethal retaliation: The dominant reason for avoiding care was the acute fear that seeking help would trigger lethal retaliation from their partner. For injuries sustained during strangulation-related assaults, concerns about the consequences of returning home to the perpetrator of NFS after a hospital visit frequently deterred women from seeking care.

*“I was scared… That he’ll do worse.”*

This fear created a harrowing calculation: the immediate medical risk of the strangulation injury was often weighed against the near certainty of escalated violence upon return.

*“I just didn’t—cause I didn’t feel safe…Cause if I went to the hospital, he probably would have jumped on me again.”*

*“If I go get help and he find out, he going to do it again, I might die or something.”*

Perceived system futility: Women believed institutional systems would not provide meaningful protection or lasting help for strangulation injuries, viewing the emergency department as a temporary, ineffective stopgap.

*“No, you don’t get help at emergency, so you just stay where you are, try to struggle it out.”*

Normalization and minimization of lethal risk: Cumulative experiences of violence appeared to shape how women interpreted strangulation, often diminishing the perceived severity of the event despite potentially life-threatening consequences. One participant, who lost consciousness during a strangulation assault, reflected:

*“It started off with a little choke. When I came back to, stars leave my face, my body’s just lingering, just weak, I’ll come back to and say, ‘Please, can you please stop choking me. I can’t breathe!’”*

Women’s descriptions of strangulation frequently contrasted with the calculated manner in which it was used by their partners. As a mechanism of coercive control, strangulation was often employed to assert dominance and instill fear, yet women sometimes recounted these experiences in understated terms. One participant explained:

*“It’s just choking. I don’t know how to even—if that’s easiest way—I don’t know if you want to say on a floor, in a corner or—it’s choking and strangulation, it’s the same—you know, sometimes the incidents did happen.”*

Another woman’s account demonstrated the intentionality underlying the violence, explaining how her resistance was met with the use of strangulation as a calculated escalation designed to reassert control:

*“When he realized like, ‘Dang, she knows how to fight,’ he would choke me out… So when he realized, this was the tactic: ‘Okay, when I attack her, she knows how to fight. I need to choke her and make her pass out.’”*

Trauma of recall: The severity of strangulation was reflected in the emotional difficulty women experienced when recounting these events. At times, interviews were paused or ended due to distress. One participant stated:

*“I hate talking about it. I want to put it behind me where I’m on the other side of the table, where I’m not crying when I talk about it. I don’t want to cry anymore. I don’t want to hurt no more. I want to be on the other side of the table asking young women, ‘What do you think you can do to survive? How can we help you?’”*

Polyvictimization and cumulative trauma: Strangulation rarely occurred in isolation. Rather, women described IPV as encompassing multiple, intersecting forms of violence that often occurred simultaneously or accumulated over time. Within these broader patterns of abuse, strangulation was experienced alongside other severe forms of harm, compounding both trauma and shame. Survivors’ narratives revealed experiences in which multiple forms of violence co-occurred within relationships and accumulated across the life course. This complexity is illustrated in the following accounts. One woman condensed the overlapping horrors of a single IPV incident, describing how multiple forms of violence converged in a single episode:

*“I was choked, beat, raped, all of it. It was horrible.”*

Another woman’s history illustrated how trauma compounds over a lifetime, beginning with a violent branding:

*“He wasn’t the first person that did it. The first guy was my first boyfriend when I was 21. He tatted my neck… he put his name on my neck, because I was young, and he was an older guy. He was 31. That was my first abuse. I was strong enough to get away from him to end it…That’s what’s on my neck now. I’ve been trying to get it covered up for the longest…”*

This participant detailed subsequent partners victimizing her, with one even causing a miscarriage:

*“The second one was the guy that I lost the baby with, and I believe he was the cause of me losing the baby because he kicked me in a corner before I even knew I was pregnant and the baby was gone. The third was my daughter’s father, so I’ve actually been doing three, but the pandemic one is really the worst one…”*

Together, these women’s narratives show how NFS is embedded within and intensified by a broader ecology of violence.

## 4. Discussion

### 4.1. Black Women’s Experiences of NFS and System Navigation

The voices of Black women who have experienced NFS add an essential and previously underrepresented contribution to the science. Guided by BFT, our research deepens understanding of Black women’s experiences of NFS and their efforts to seek safety and care following violence. Their accounts provide knowledge that clinical indicators alone cannot capture, revealing how NFS is experienced, recognized, described, and understood within the context of IPV. Importantly, their experiences also illuminate how structural racism and experiences of discrimination within healthcare can shape whether healthcare is perceived as a safe place to seek care following violence. For some Black women, these experiences may contribute to mistrust, avoidance, or reluctance to disclose violence, creating barriers to healthcare access when assessment and intervention may be most critical to their survival. By centering survivors’ voices, this study expands the evidence base beyond the presence or absence of visible injury and demonstrates that understanding NFS requires attention not only to its physical consequences, but also to the structural and interpersonal conditions that shape Black women’s pathways to safety, healthcare, and support.

Structural racism emerged as a primary barrier to Black women’s pathways to healthcare following NFS. Women’s experiences illustrate how these broader structural inequities may manifest within healthcare encounters, influencing whether their experiences were recognized, validated, and met with appropriate care and support. The women’s concerns about seeking care were shaped not only by their prior experiences, but also by an awareness of the historical mistreatment of Black people in United States history, including the exploitation and nonconsensual experimentation on Black bodies and the use of Black women in medical procedures without consent [36]. In addition to these historical transgressions, the women’s previous harmful interactions with healthcare professionals contribute to a broader context in which Black women may anticipate discrimination, dehumanization, disbelief, or inadequate treatment when seeking healthcare [11,24]. Such anticipation is consequential because when healthcare was not perceived as a safe or trustworthy space, the women delayed or avoided care following violence, potentially limiting opportunities for timely assessment of NFS and other injuries. These findings underscore the need for culturally responsive care education across the healthcare workforce.

Our findings also point to the importance of material constraints operating alongside racism and gendered violence. Half of participants were unemployed (50%), and among those who reported annual household income, most reported incomes below $20,000, including public assistance, reflecting substantial economic disadvantage among participants with available income data. These economic circumstances are especially important when considering NFS, a form of IPV with heightened risk for severe and lethal violence. Economic precarity, housing instability, and financial dependence can constrain survivors’ ability to leave abusive relationships, secure alternative housing, access healthcare, or engage with formal systems following IPV [37]. For Black women, these constraints may be compounded by structural and institutional racism, which further shapes perceptions of whether formal systems are accessible, trustworthy, or safe [9,16,37,38]. Thus, economic precarity should not be understood solely as an individual financial barrier, but as an intersecting structural condition through which racism, sexism, and socioeconomic position may shape Black women’s options for seeking safety after incidents of NFS and other forms of IPV [21].

### 4.2. Implications for Professional Education and System Practice

Education on culturally responsive, trauma- and violence- informed care should extend beyond emergency department (ED) nurses and physicians to professionals across healthcare and victim-serving systems, including primary and urgent care professionals, forensic nurse examiners (FNEs), advocates, and law enforcement officers. Women’s decisions to seek help were shaped by cumulative trauma and previous harmful institutional experiences, which, viewed through a BFT lens, may reinforce self-reliance as a strategy for managing violence and limiting reliance on formal systems [19,21]. Women also described shame, humiliation, and public exposure following contact with law enforcement, demonstrating how systems’ responses intended to provide protection can instead compound harm. These findings emphasize the importance of ensuring privacy, safety, dignity, and emotional support across points of system contact [39].

Trauma- and violence-informed care (TVIC) provides a framework for addressing these concerns by recognizing the effect of interpersonal and structural violence and prioritizing physical, emotional, and cultural safety, collaboration, choice, and survivors’ strengths [40,41,42]. Recommendations for trauma-informed, survivor-centered care extend beyond the United States, with the World Health Organization and Australian health and family violence organizations providing recommendations for trauma-informed, survivor-centered care for individuals experiencing violence [43,44]. Importantly, TVIC places responsibility on organizations and professionals to address policies and practices that may perpetuate harm rather than requiring survivors to navigate complex systems or repeatedly disclose their experiences [41]. For Black women survivors, culturally responsive and trauma- and violence-informed practices may be particularly important when prior experiences of discrimination or institutional harm shape perceptions of healthcare as unsafe. Professional education should therefore prepare providers across systems to recognize these dynamics, respond without judgment, and create safe environments in which survivors can seek care and support without anticipating further harm.

### 4.3. Lethality and Safety

Concurrently, when survivors perceive that their experiences will not be believed or taken seriously, they may delay seeking assistance or avoid relying on formal systems until the threat of violence has escalated to a critical or potentially lethal level [24]. This pattern is particularly concerning for Black women survivors given the disproportionate burden of lethal violence among Black women and the increased risk of IPV-related lethality associated with NFS. National data demonstrate persistent inequities in homicide, with Black women experiencing substantially higher homicide rates than White women; these disparities are especially pronounced in the Upper Midwestern state in the United States where participants were recruited, where Black women aged 22–44 years were approximately 20 times more likely to die by homicide than White women in 2019–2020 [45]. Research examining IPV lethality risk among Black women further suggests that experiences of discrimination are associated with higher Lethality Assessment Program (LAP) scores [46]. Together, these findings underscore the importance of early identification of lethality risk and safety planning that accounts for the intersecting risks experienced by Black women survivors. NFS should be recognized as an additional and distinct marker of lethality within IPV, reinforcing the need for timely screening, assessment, and intervention.

IPV lies at the intersections of healthcare and legal systems, positioning law enforcement officers, advocates, and healthcare professionals as critical stakeholders in identifying, responding to, and supporting survivors across multiple points of system contact. Findings from our study have two important implications for practice. First, systematic lethality assessment and safety planning are needed across systems that respond to IPV. Law enforcement officers are commonly the first professionals to assess immediate safety and connect survivors with resources, yet responses do not always meet survivors’ needs [47,48]. The LAP provides an evidence-based approach to identifying survivors at elevated risk for severe or lethal violence and facilitating connection with domestic violence advocates [49]. In the Upper Midwestern state where participants were recruited, 42 of 72 counties have at least one law enforcement officer trained in LAP, although statewide LAP implementation is not mandated [50]. Expanding statewide implementation could promote more consistent assessment of lethality risk and access to advocacy and safety resources across jurisdictions. For survivors identified as high risk, coordinated responses among law enforcement, advocates, emergency medical services (EMS), and healthcare professionals are important to support timely safety planning and evaluation of potentially life-threatening injuries.

Second, identification of IPV should prompt assessment of NFS. Healthcare professionals, including ED nurses, physicians, and FNEs, may be among the first healthcare providers to encounter survivors following IPV. NFS assessment should extend beyond simply asking whether strangulation has occurred to considering how questions are asked. TVIC and culturally responsive care require clinicians to use language that survivors recognize and understand, such as *choking*, rather than relying exclusively on the clinical term *strangulation*. For example, clinicians might ask, “Has your partner ever choked you?” followed by behaviorally specific questions such as, “Has your partner ever put pressure on your neck or chest with their hands, knee, or body weight?” Such questions may facilitate disclosure when survivors do not identify their experiences using the term strangulation. Incorporating routine NFS assessment following a positive IPV screen may therefore improve identification of this serious form of violence and create opportunities for timely medical evaluation, safety planning, and referral.

Clinical guidance emphasizes the importance of systematic assessment and management of NFS in acute and emergency care settings. Recommendations from the Training Institute on Strangulation Prevention (2022) provide guidance for medical and radiographic evaluation based on patients’ physical assessment findings, history of the assault, and reported symptoms, while clinical guidelines from the Institute for Addressing Strangulation in the United Kingdom and the Emergency Care Institute in Australia provide recommendations for assessment, imaging, management, discharge, referral, and follow-up [51,52]. Significantly, identification of NFS should prompt a focused clinical assessment rather than serve as the endpoint of screening. Following a positive NFS screen, ED providers should assess for symptoms and signs of potentially serious injury, recognizing that significant internal injury may occur in the absence of visible external trauma [53]. Reliance on visible injury may be particularly problematic when assessing patients with darker skin tones, as external injuries may be less readily recognized or documented, underscoring the importance of incorporating survivors’ reported symptoms and the mechanism of injury into clinical assessment [54,55]. Together, these recommendations underscore that effective NFS care requires more than identification of strangulation; it requires timely, symptom-informed clinical evaluation supported by provider knowledge, appropriate diagnostic resources, and systems prepared to respond to survivors’ needs.

### 4.4. Research Implications

Future research should continue to center Black women’s voices in studies of IPV, NFS, healthcare access, and lethality risk. Research is needed to examine how Black women describe and recognize NFS, how language influences disclosure, and whether routine NFS assessment following a positive IPV screen improves identification and timely care. Studies should also evaluate implementation of consistent LAP to determine whether systematic lethality assessment improves safety planning and access to resources for Black women survivors. Importantly, future research should examine how structural racism, discrimination, and conditions of living intersect with repeated NFS and cumulative trauma to shape lethality risk, help-seeking, and pathways to safety. Survivor-informed intervention research that moves beyond individual screening to address organizational and system-level responses is needed to develop more equitable, trauma-informed approaches to recognizing risk, responding to violence, and supporting survivors.

### 4.5. Study Limitations

There are a few limitations of our study that should be noted. First, participants were recruited from a single urban region in an upper Midwestern state during the COVID-19 pandemic; therefore, the findings may not reflect the experiences of Black women in other geographic regions or time periods. Second, although experiences of NFS emerged as a prominent theme across interviews, the broader study was designed to explore experiences of intimate partner violence and help-seeking rather than strangulation specifically. As a result, participants were not asked a standardized set of questions about strangulation, and some experiences may not have been fully explored. Third, all data were self-reported and are subject to recall bias. The retrospective nature of these accounts also introduces the possibility of recall limitations, particularly for events that occurred further in the past. Trauma may influence how experiences are remembered and narrated; therefore, the analysis did not attempt to establish precise event chronology when participants had not provided it. Rather, accounts were interpreted as survivors’ descriptions and meaning-making of their experiences at the time of interview. Given the traumatic nature of violence and strangulation, some participants found recounting these experiences distressing, which may have influenced the depth of information shared. Finally, the parent study was not designed to examine how sexual orientation, gender identity, or involvement in informal or criminalized survival economies may shape experiences of NFS and healthcare access. These intersecting social positions may create additional barriers to disclosure, institutional trust, and healthcare access that were not captured in the present analysis and warrant further investigation. Despite these limitations, this study provides important insights into how structural racism, shame, and fear shape healthcare access among Black women survivors of NFS and highlights critical implications for forensic nursing practice and survivor-centered care.

## 5. Conclusions

Nonfatal strangulation is one of the most lethal forms of intimate partner violence, yet it remains frequently overlooked within healthcare and legal systems. For the Black women in this study, decisions to seek care after strangulation were shaped not only by the severity of the assault but also by structural racism, shame, fear of retaliation by abusive partners, and prior experiences of institutional harm. These findings demonstrate that barriers to care extend beyond individual help-seeking decisions and are embedded in broader social, historical, and structural inequities. Healthcare providers and systems must recognize nonfatal strangulation as a critical opportunity for intervention while addressing the racism, stigma, and institutional mistrust that discourage survivors from seeking care. Ultimately, improving outcomes for Black women survivors requires more than enhancing strangulation assessment and lethality screening. It requires creating safe post-assault care that survivors perceive as physically, emotionally, and culturally safe; that affirms their dignity; that acknowledges the realities of structural racism and cumulative trauma; and that is worthy of their trust. Building such systems through trauma- and violence-informed, culturally responsive care and meaningful partnerships with survivors and communities is essential to reducing disparities and improving outcomes following one of the most lethal forms of intimate partner violence.

## Figures and Tables

**Table 1:** Participant Characteristics (*n* = 28).

Demographic Variable	Mean (SD) or *n* (%)
Age	37.18 (9.63)
Education Level	
Less than high school	6 (21.4)
High school diploma	15 (53.6)
Some college but no degree	2 (7.1)
Bachelors degree	4 (14.3)
Missing/not reported	1 (3.6)
Employment Status	
Yes	14 (50.0)
No	14 (50.0)
Annual Household Income	
<$10,000	7 (36.8)
$10,000–$19,999	5 (17.9)
$20,000–$29,999	5 (17.9)
$30,000–$39,999	2 (7.1)
Missing/not reported	9 (32.1)

## Data Availability

All relevant qualitative data supporting the conclusions of this study are contained within the article. Full raw interview transcripts are not publicly available due to participant privacy and ethical restrictions regarding sensitive human subject data.

## Abbreviations

The following abbreviations are used in this manuscript:

IPV	Intimate partner violence
NFS	Nonfatal strangulation
CAB	Community advisory board
PTSD	Posttraumatic stress disorder
ED	Emergency department
BFT	Black Feminist Thought
EMS	Emergency medical service
FNE	Forensic Nurse Examiner
